# Supplementary material for: Machine learning-based framework for wall-perching prediction of flying robot
Source: Nat Commun. 2025 Dec 11;16:11038. doi: 10.1038/s41467-025-67386-0 (PMC12698687; doi:10.1038/s41467-025-67386-0)
Supplement: Supplementary file 1 — Supplementary Information [file 41467_2025_67386_MOESM1_ESM.pdf]

Supplementary Information for

Machine learning-based framework for wall-perching prediction of flying robot

Yunian Shen[1]*†, Chenxi Mao[1]†, Zeyu Qi[1], Kunpeng Liu[1], Weixu Zhang[1], An Cao[1]

[1]Department of Mechanics and Engineering Science, School of Physics, Nanjing University of Science and Technology; Nanjing, 210094, P. R. China.

*Corresponding author. Email: yunianshen@njust.edu.cn

†Co-first author

This file includes:

Supplementary Notes 1 to 3

Supplementary Figures 1 to 9

Supplementary Tables 1 to 3

Supplementary References

Supplementary Note 1: Detailed descriptions of attachment system to climb the wall

(1) Wall-climbing mechanism

Supplementary Fig. 1 shows the pose schematic of the attachment system, illustrating the positions of its joints and links, along with their degrees of motion freedom. The wall-climbing mechanism primarily consists of two symmetrical spines, two steering gears, two flexible nylon cables, a rigid transverse carbon fiber rod, and a T-shaped rod. In each spine, one end of the flexible slender carbon fiber crank is connected to the frame via a link, while the other end connects to the spine. By tightening the nylon cable, the flexible rod is tensioned into an arc shape. One end of the nylon cable connects to the spine, and the other end attaches to the transverse carbon fiber rod. The mid-sections of the two nylon cables pass through the left and right small holes at the ends of the T-shaped rod, respectively. The two steering gears are the spine extension or retraction control steering gear and the spine lift or lower control steering gear. The transverse carbon fiber rod is driven to rotate by the extension or retraction control steering gear, while the T-shaped rod is driven to rotate by the lift or lower control steering gear. This configuration ensures the overall structure meets stability requirements.

The extension and retraction motion of the spine is achieved by the extension and retraction servo motor driving the transverse straight rod to rotate around the θ_1 axis. This rotation tightens or loosens the corresponding side's nylon rope, utilizing the elastic restoring force of the flexible curved rod to drive the spine's extension or retraction. The lifting and lowering motion is achieved by the lifting and lowering servo motor driving the T-shaped rod to rotate around the θ_2 axis. This rotation causes the two ends of the T-shaped rod to move in opposite directions. Through transmission via nylon ropes and nylon pulleys, this motion lifts or pulls down the spine, completing the foot lifting and placing actions.

The geometric relationship between the components in the attachment system is expressed as:

$$s' = \sqrt{(l_1 + l_2)^2 - (l_1 + l_2)d_1 \cos\theta_1 - \frac{d_1 d_2}{2}\cos\theta_2 \sin\theta_1 + \frac{\left(d_1^2 + d_2^2\right)}{2}} \tag{1}$$

Among these, s' is the length of the rope from the end point of the straight rod to the end point of the T-shaped rod, l_1 is the length from the end point of the horizontal rod to the end point of the curved rod when the horizontal rod and the T-shaped rod are perpendicular, l_2 is the length from the end point of the curved rod to the end point of the T-shaped rod, d_1 is the length of the horizontal rod, and d_2 is the width of the T-shaped rod. The length of the rope from the end point of the T-shaped rod to the claw tip is s-s'. The specific position of the claw tip is related to the deformation of the flexible rod and requires dynamic calculations to obtain the deformation of the flexible rod. Furthermore, the length of l_3 and the size of the stride can be adjusted using Supplementary Equation (1).

(2) Spine structure

The robotic spine is the core structure for physical adhesion in spine-based wall-climbing robots. This paper draws inspiration from the design proposed by Parness et al.[1] for NASA's Mars rover and further improves upon it, resulting in a smaller and more refined spine structure. As shown in Supplementary Fig. 2a, the spine structure consists of four components: a spine holder, a curved rod connector, a pin, and the spine tip.

Since the spine is a passive component, it must transmit both the pulling force from the nylon rope and the restoring force from the carbon fiber rod, while ensuring its motion trajectory closely aligns with the desired path. To restrict the spine's movement to a planar motion in the vertical space, we introduced a spine holder and a curved rod connector as shown in Supplementary Fig. 2b. By increasing the number of necessary components, the degrees of freedom of the spine in

spatial motion are effectively constrained, enabling more stable surface adhesion.

Both the spine holder and the curved rod connector are made of resin material through mold casting. The spine is embedded and fixed within the spine holder, while the spine holder and the curved rod connector are securely connected via a pin. One end of the spine holder is attached to the nylon rope, and the other end is linked to the curved rod connector, making it a two-force member. Its position is determined by the direction of the nylon rope's pulling force and the configuration of the carbon fiber rod. Since the applied flexible rope tension and the carbon fiber rod's configuration lie in the same plane, the displacement of the spine holder perpendicular to this plane is negligible. Therefore, it can be assumed that the spine remains in planar motion during climbing. Additionally, during foot placement and lifting, the spine only undergoes fixed-axis rotation around the pin.

Supplementary Note 2: Details of the landing experiment

Here we provide further details on the landing experiment mentioned in the main text. We built a complete experimental system (Supplementary Fig. 3) consisting of a robot, a data-acquisition and control module, and a video-recording module. The data-acquisition and control module includes a Windows-based laptop and an Xbox controller. They command the robot’s flight and landing maneuvers while simultaneously gathering its state parameters through sensors. The video-recording module comprises auxiliary lighting and an HD video camera to capture visual data during the landing phase. The robot’s autonomous control software (Supplementary Fig. 4) was developed in-house in Python on top of the Crazyflie client and runs on the laptop. It comprises three control sub-programs:

1) State data computation sub-program. It is used to record the data of the robot's sensors. The quadrotor communicates with the PC using the Crazyradio PA 2.4 GHz USB dongle to transmit the data obtained from the acceleration sensor and gyroscope wirelessly. Additionally, the sub-program performs calculations on the collected data to generate the required input for the machine learning framework.

2) Landing sub-program 1-contacting detection. If a successful landing is predicted in advance, this program will be used to detect whether the robot makes contact with the wall or not.

3) Landing sub-program 2-flips upward controlling. This program is used to control the robot to perform upward flips by adjusting the brushless motor.

An experiment was conducted involving a spine bio-inspired robot landing on a vertical wall. In this experiment, a high-density sponge mat with a surface hardness approximately equivalent to that of soft bark was used as the wall. Initially, the robot was manually operated using an Xbox One pad to control its flight towards the vertical wall. Once the robot detected contact with the wall using the "contacting detection program", the Crazyflie client would automatically take over and operate the robot. The Crazyflie client sends commands to control the motor, allowing the robot to successfully land and subsequently perch on the vertical wall. Throughout the entire landing process, an HP Zhan66 laptop with the Crazyflie client is used to record all data from the BMI088 MPU-9250 3-axis gyro sensor at a frequency of 50Hz. The entire landing process is captured in an image with a resolution of 1920×1080 pixels, recorded by a Sony HDR-CX680 camera.

Based on the simulation results, it is evident that if the robot's initial velocity is less than or equal to 0, it will be unable to collide with the wall. Conversely, an excessively high initial velocity can cause damage to the landing system. Therefore, it is recommended to set the robot's initial

velocity between 0 and 1.5m/s. Moreover, when the velocity of the spines upon impact with the surface is too high, it becomes challenging to achieve attachment and may even result in damage to the attachment mechanism too. Hence, the velocity of the spines during landing should not exceed roughly 5m/s. In the case of a quadrotor aircraft, it is necessary to lean forward to generate a forward component of lift, which leads to stable forward flight with a pitch angle less than 0. Consequently, the recommended range for the robot's initial angle is between 0 and $-15°$.

Supplementary Note 3: The results of multi-layer perceptron (MLP), Support Vector Machine (SVM), and random forest (RF)

To compare the prediction results of different methods for this research content, we employed three machine learning methods, namely MLP, SVM, and RF, to make predictions for this study.

(1) MLP

A deep learning program based on the TensorFlow software package was developed. The dataset used was consistent with the mixed datasets mentioned in the manuscript. Before the model training, the mixed dataset was randomly divided into the training set and the test set in an 8:2 ratio. The overall process of the program is shown in Supplementary Fig. 7e.

Since this research is a binary classification problem, a MLP model consisting of five fully connected layers was constructed. The activation functions of the hidden layers all adopted ReLU, and the activation function of the final output layer was sigmoid. The Adam optimizer was selected, and the binary cross-entropy was taken as the loss function, while the accuracy rate was monitored. Since the dataset of this experiment is small, full-batch gradient descent is adopted for model training. Meanwhile, 10% of the training data is divided into the validation set to verify the model performance.

The training results are shown in Supplementary Fig. 7. The training results indicate that when the training reaches around 100 generations, the loss function has approached 0 and remained stable. This suggests that the model has effectively learned the patterns and features in the data, achieving high accuracy and good fitting results on the training data.

To intuitively present the results of MLP prediction, decision boundaries for predicting whether the robot can land successfully were generated based on the trained model with the initial angle as the abscissa and the initial velocity as the ordinate. To ensure the accuracy of the decision boundaries, the prediction mesh is selected as 500×500. The generated decision boundaries were shown in Supplementary Figs. 7a and 7b.

(2) SVM

A SVM algorithm implemented using the LIBSVM library was developed for prediction success of landing. The dataset remained consistent with that described in the manuscript. The overall process of the program is shown in Supplementary Fig. 8e.

Given the binary classification nature of this research, a radial basis function (RBF) kernel was selected for the SVM. To optimize model performance, a systematic grid search approach was employed to identify the optimal hyperparameters – the regularization parameter *c* and kernel coefficient γ. This search methodically evaluated parameter combinations across logarithmically scaled intervals (*c*: [$2^{5,}$ 2^{15}], γ: [2^{-15}, 2^{3}]). Besides, Leave-one-out cross-validation (LOOCV) was rigorously applied during training to ensure robust generalization and prevent overfitting, leveraging each data point iteratively as a validation sample. Model performance was assessed using classification accuracy.

The training results are shown in Supplementary Fig. 8. Among them, the result for the "Lift receding" situation are as follows: parameter *c* is 362.0387, parameter γ is 0.3536, and the cross-

validation accuracy rate reaches 99.10%. The result for the "Lift strengthening" situation is as follows: parameter c is 128, parameter γ is 0.1768, and the cross-validation accuracy rate reaches 95.61% (see Supplementary Table 2).

To visualize the decision-making mechanism, the initial Angle is used as the abscissa and the initial velocity as the ordinate, and a 500×500 grid is adopted to depict the predicted boundary. The results are shown in Supplementary Figs. 8a and 8b.

(3) RF

RF model is established based on the mixed dataset, and the main parameter adjusted is the number of decision trees. The overall process of the program is shown in Supplementary Fig. 9d. The curve of the accuracy rate varying with the number of decision trees is shown in Supplementary Fig. 9c. According to leave-one-out cross-validation, for the case of lift receding, the model's accuracy reached a peak of 94.81% when the number of decision trees was 22; while for lift strengthening, the highest accuracy of 91.14% was achieved with 10 decision trees (see Supplementary Table 2). In addition, the random sampling during the training of the random forest may lead to the omission of some key data points, thereby affecting the generalization ability of the model. Therefore, the prediction accuracy under small sample data is not as good as that of MLP. Similarly, the decision boundaries of random forests were shown in Supplementary Figs. 9a and 9b.

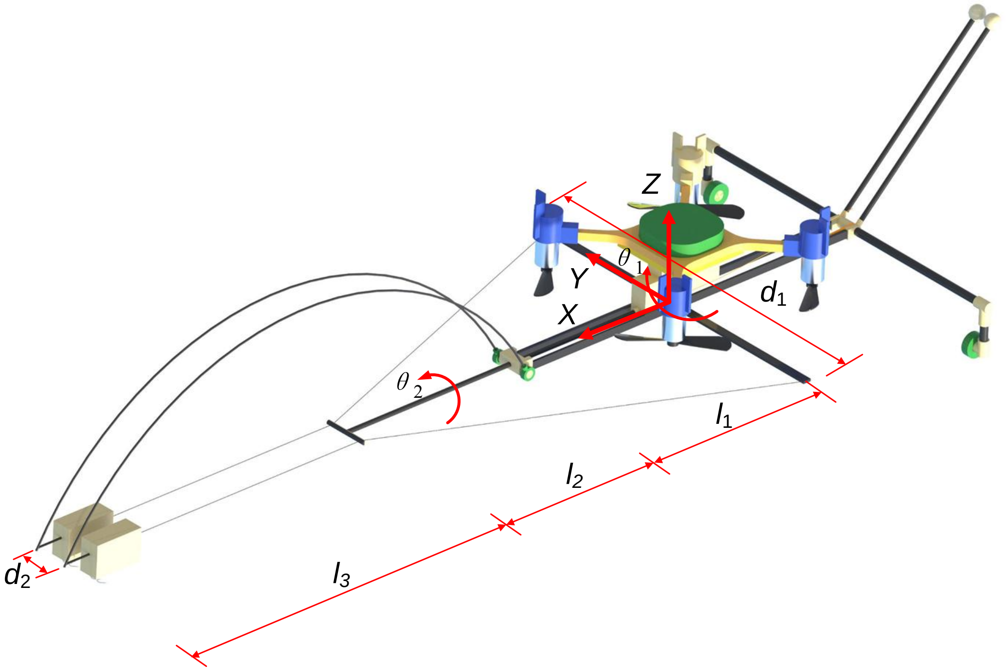

Supplementary Fig. 1 Configuration diagram of attachment system

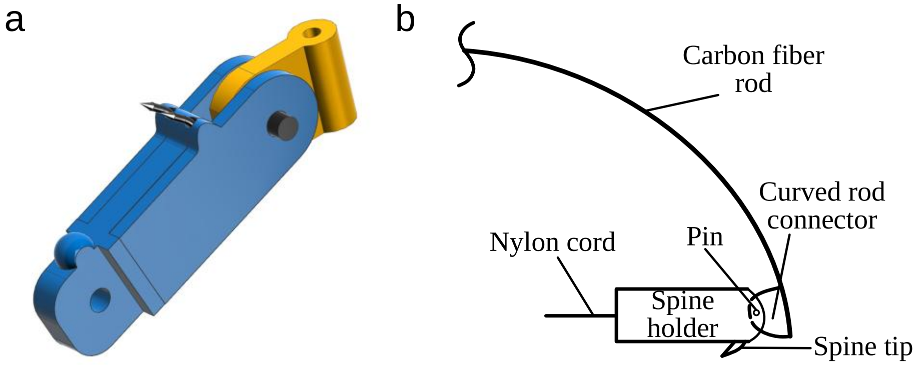

Supplementary Fig. 2 Structural diagram of the spine and its combined mechanism. a Structural diagram of the spine produced by UG software, **b** Schematic diagram of the combined mechanism of spine, carbon fiber rod and nylon cord.

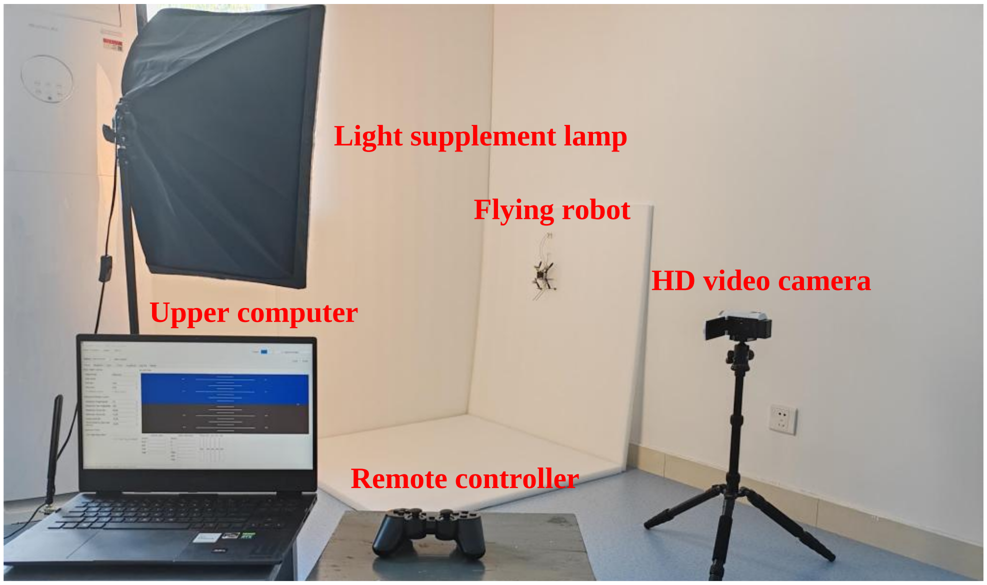

Supplementary Fig. 3. Experimental site diagram.

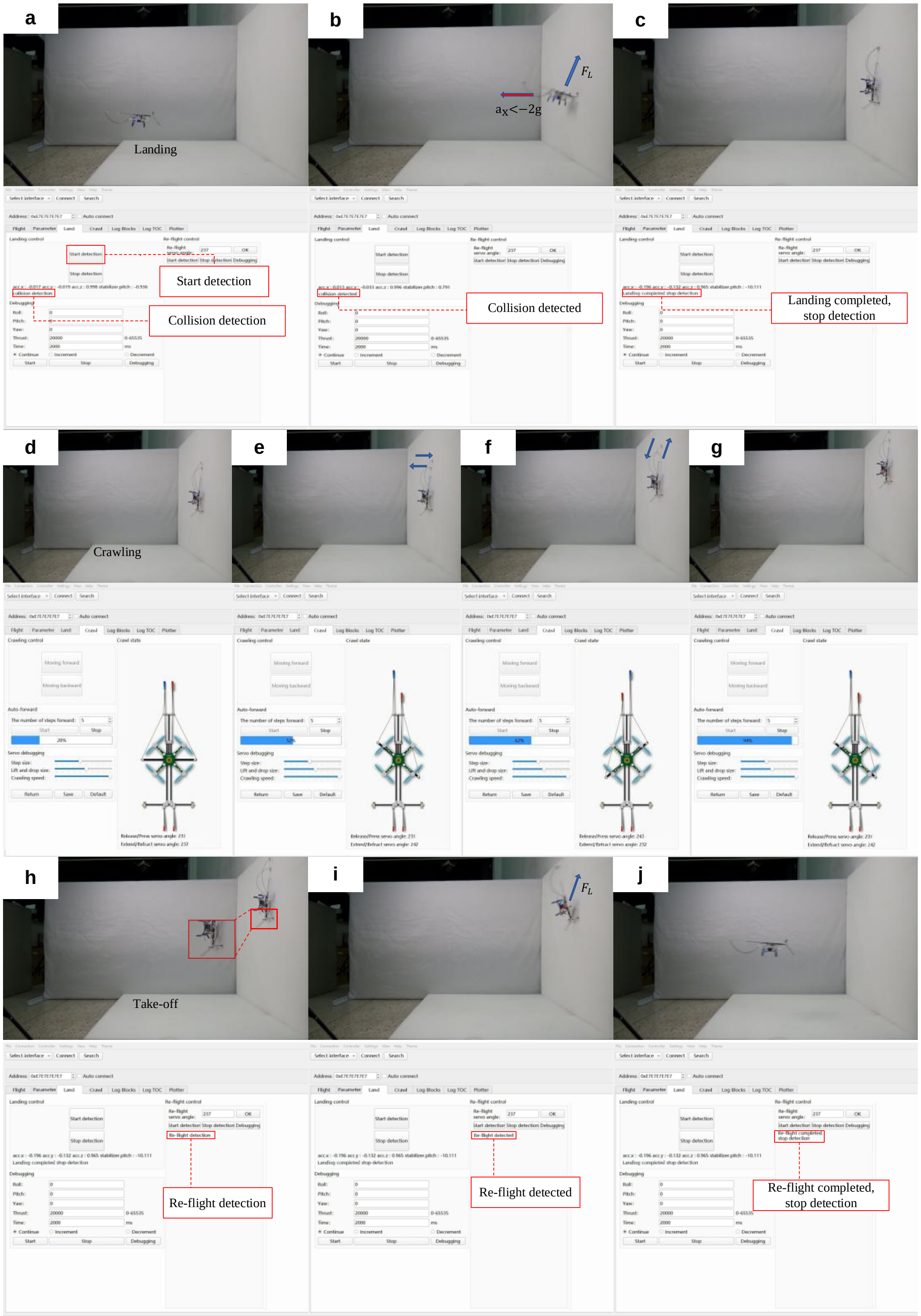

Supplementary Fig. 4. Robot experimental process and control software. a Approaching the wall. **b** Landing on the wall. **c** Landing completed. **d-g** Spine climbing motion. **h-j** Taking-off.

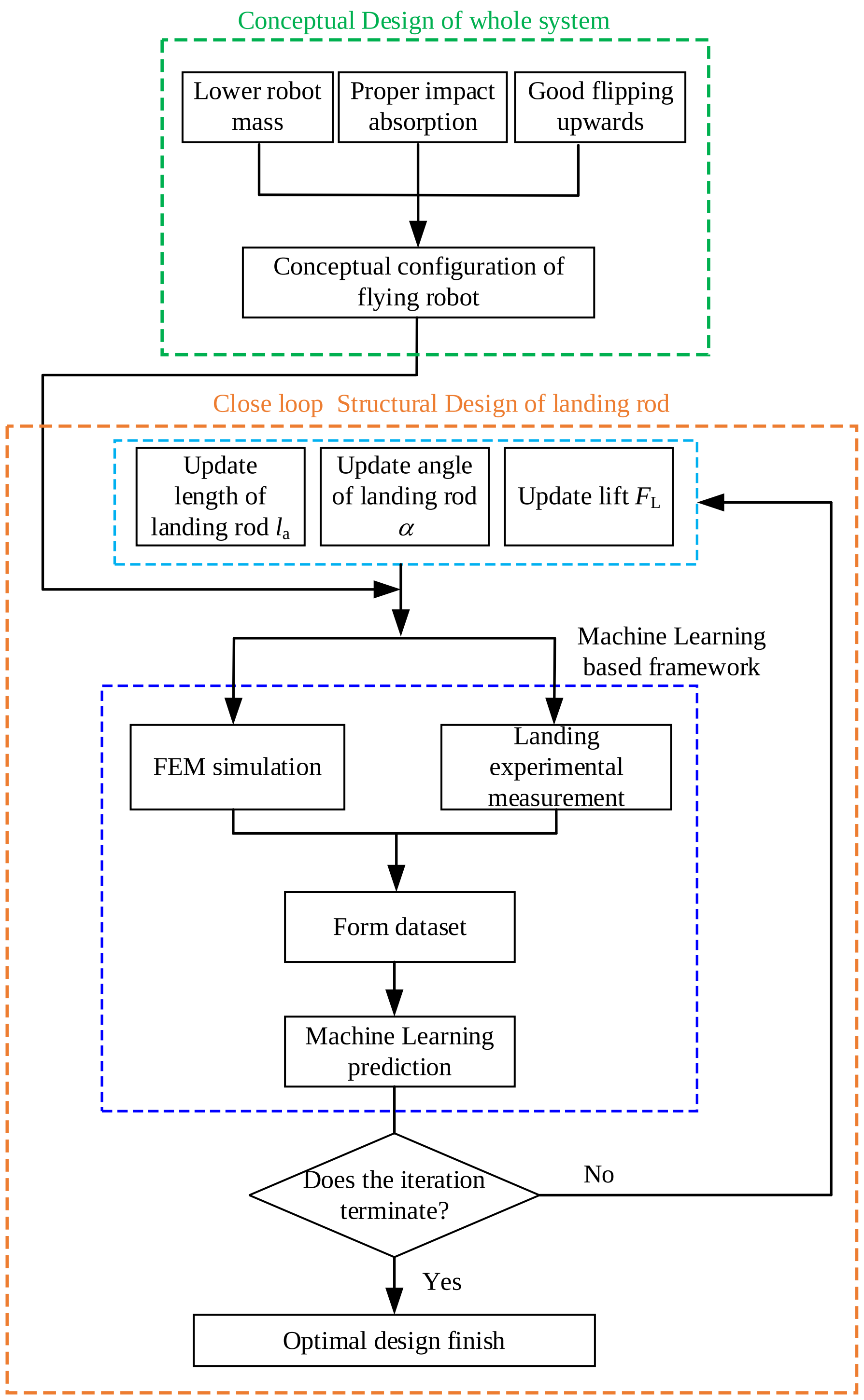

Supplementary Fig. 5. The flow chart of close loop design analysis.

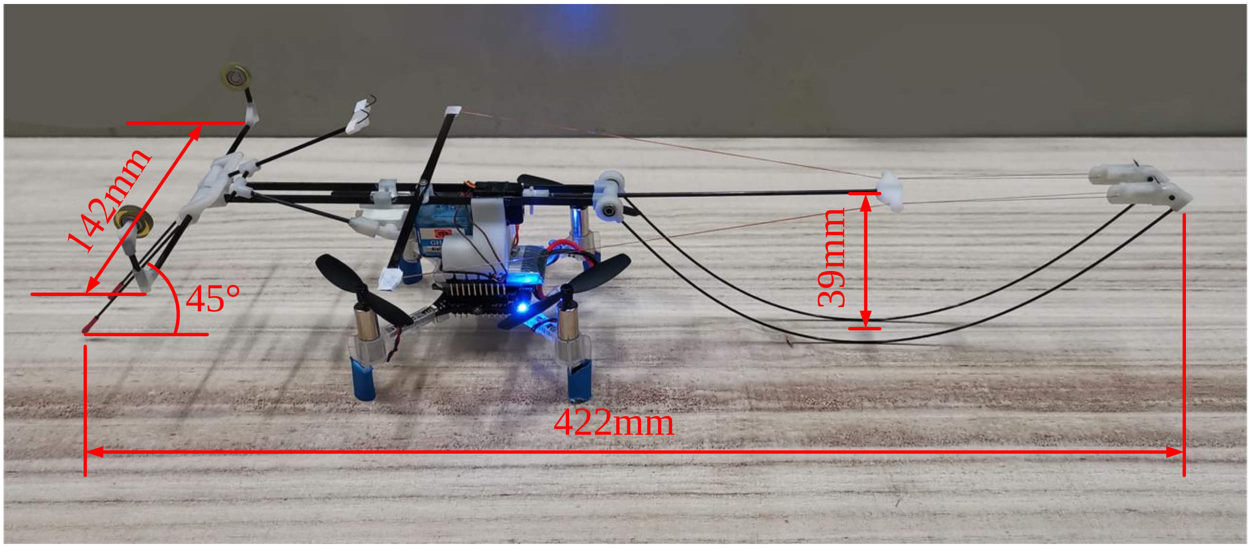

Supplementary Fig. 6. The geometric size of robot.

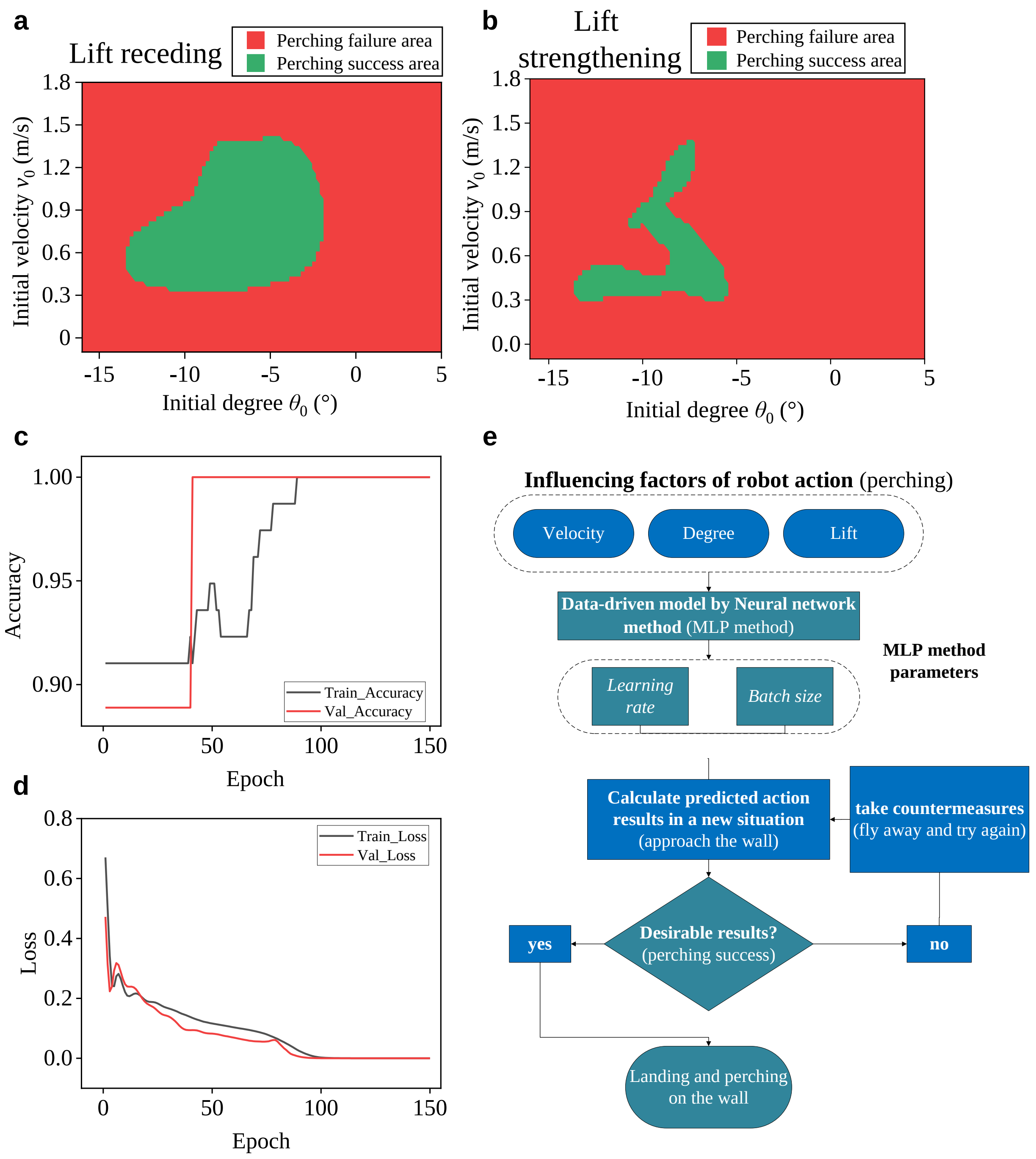

Supplementary Fig. 7. Decision boundaries and prediction accuracy by MLP model. a The decision boundary divides the data set obtained under lift receding into the perching successful and failure areas. **b** The decision boundary divides the data set obtained under lift strengthening into the perching successful and failure areas. **c** The prediction accuracy of MLP with epochs. **d**

The prediction loss of MLP with epochs. **e** The flow chart of using MLP to predict the landing behavior.

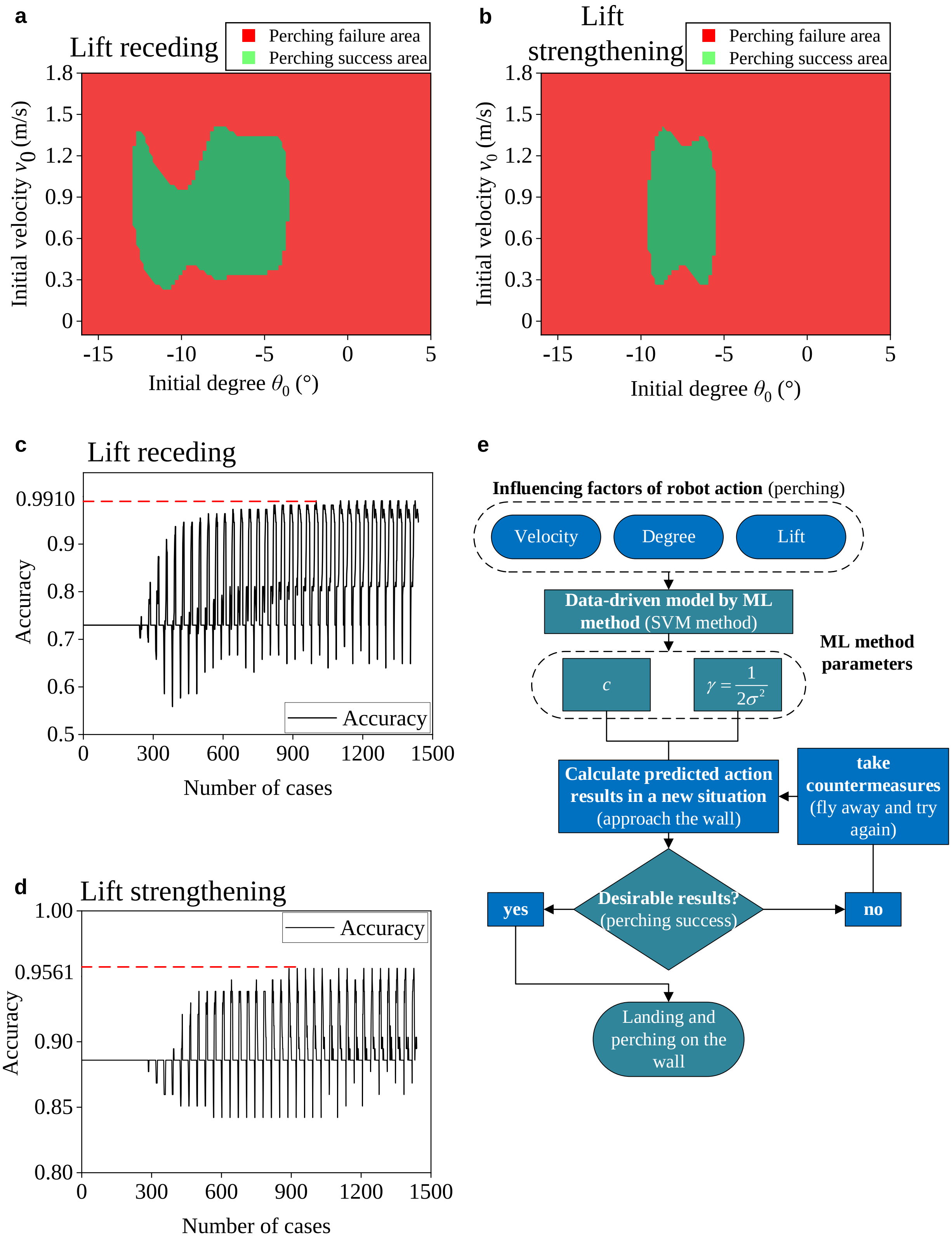
a
Lift receding
Perching failure area
Perching success area
Initial velocity v_0 (m/s)
Initial degree θ_0 (°)
b
Lift strengthening
Perching failure area
Perching success area
Initial velocity v_0 (m/s)
Initial degree θ_0 (°)
c
Lift receding
0.9910
Accuracy
Number of cases
d
Lift strengthening
0.9561
Accuracy
Number of cases
e
Influencing factors of robot action (perching)
Velocity
Degree
Lift
Data-driven model by ML method (SVM method)
ML method parameters
c
$\gamma = \frac{1}{2\sigma^2}$
Calculate predicted action results in a new situation (approach the wall)
take countermeasures (fly away and try again)
Desirable results? (perching success)
yes
no
Landing and perching on the wall

Supplementary Fig. 8. Decision boundaries and prediction accuracy by SVM model. a The decision boundary divides the data set obtained under lift receding into the perching successful and failure areas. **b** The decision boundary divides the data set obtained under lift strengthening into the perching successful and failure areas. **c** The prediction accuracy of SVM under lift receding with number of cases. **d** The prediction accuracy of SVM under lift strengthening with number of cases. **e** The flow chart of using SVM to predict the landing behavior.

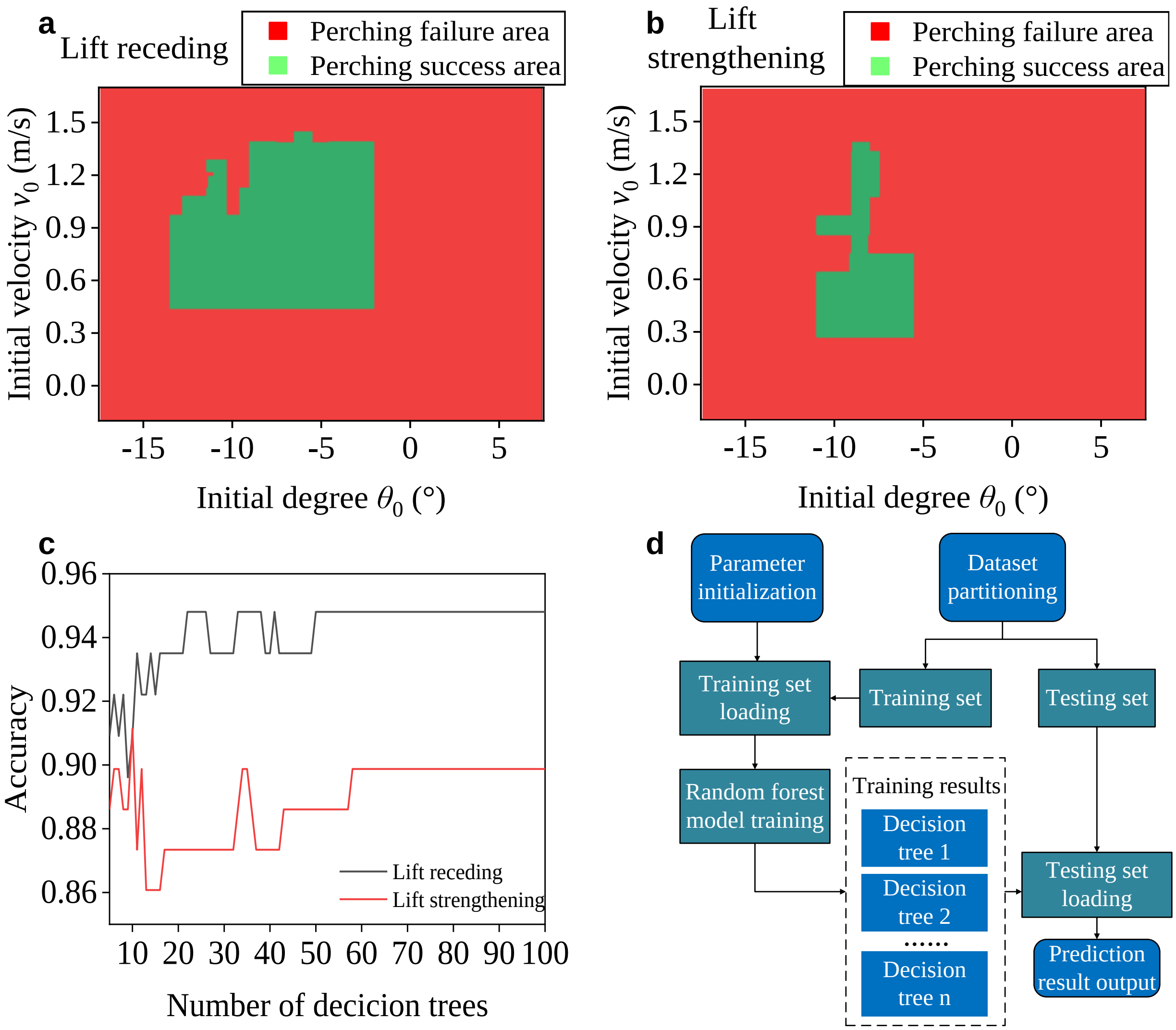

Supplementary Fig. 9. Decision boundaries and prediction accuracy by RF model. a The hyperplane divides the data set obtained under lift receding into the perching successful and failure areas. **b** The hyperplane divides the data set obtained under lift strengthening into the perching successful and failure areas. **c** The prediction accuracy of RF with the number of decision trees. **d** The flow chart of using RF to predict the landing behavior.

Supplementary Table 1. Geometric parameters of the robot.

Geometric parameters	Value
Body length	422mm
Body width	142mm
Body height	39mm
Quadcopter width	92mm
Spine tip radius	20μm
Landing rod angle	45°

Supplementary Table 2. Comparison of perching prediction between experiment data and knowledge-driven model solution.

Lift condition	Data points	Initial velocity v_0 (m/s)	Initial degree θ_0 (°)	Experimental data	knowledge-driven model solution
Lift receding	1	1.17	-11.14	●	●
	2	0.86	-7.56	○	○
	3	0.68	-10.85	○	○
Lift strengthening	4	1.13	-10.68	●	●
	5	0.63	-6.71	○	○
	6	0.49	-7.05	○	○

(○-success;●-failure)

Supplementary Table 3. Data split into testing and validation sets, and accuracy.

Method	Training data number	Prediction data number	Model	Prediction accuracy
Leave-One-Out Cross-Validation	Radom 107	remaining 1	SVM	99.10%
			RF	94.81%
	Radom 116	remaining 1	SVM	95.61%
			RF	91.14%

Supplementary References

1. Parness A. et al. Gravity-independent Rock-climbing Robot and a Sample Acquisition Tool with Microspine Grippers. *J. Field. Robot.* **30(6)**: 897-915 (2013).
