# Supplementary material for: Machine learning-based framework for wall-perching prediction of flying robot
Source: Nat Commun. 2025 Dec 11;16:11038. doi: 10.1038/s41467-025-67386-0 (PMC12698687; doi:10.1038/s41467-025-67386-0)
Supplement: Supplementary file 2 — Description Of Additional Supplementary File [file 41467_2025_67386_MOESM2_ESM.pdf]

Description of Additional supplementary files

Supplementary Movie 1. This file is experiment video of the robot landing on the vertical surface, including four types of landing behaviors.

Supplementary Data 1. This file is dataset for machine learning. The columns are: landing outcome (success: 1, failure: -1), initial angle, initial horizontal velocity, and lift adjustment (receding: 0, strengthening: 1).
